# Quality of Life, Physical and Mental Health of Family Caregivers of Dependent People with Complex Chronic Disease: Protocol of a Cohort Study

**DOI:** 10.3390/ijerph17207489

**Published:** 2020-10-15

**Authors:** Raquel Marfil-Gómez, Marta Morales-Puerto, Álvaro León-Campos, José Miguel Morales-Asencio, Juan Carlos Morilla-Herrera, Eva Timonet-Andreu, Magdalena Cuevas-Fernández Gallego, Celia Martí-García, Inmaculada López-Leiva, Silvia García-Mayor

**Affiliations:** 1 Department of Nursing, Faculty of Health Sciences, University of Málaga, 29017 Málaga, Spain; raquelmg1994@gmail.com (R.M.-G.); jmmasen@uma.es (J.M.M.-A.); jmorilla29@gmail.com (J.C.M.-H.); magdacuevas@gmail.com (M.C.-F.G.); celiamarti@uma.es (C.M.-G.); mainma@uma.es (I.L.-L.); sgmayor@uma.es (S.G.-M.); 2 Agencia Sanitaria Costa del Sol. Marbella, 29651 Málaga, Spain; martastuart16@gmail.com (M.M.-P.); evatimonetandreu@gmail.com (E.T.-A.); 3 Instituto de Investigación Biomédica de Málaga (IBIMA), 29010 Málaga, Spain; 4 Servicio Andaluz de Salud (SAS), Distrito Sanitario Málaga—Valle del Guadalhorce, 29009 Málaga, Spain

**Keywords:** nurses, nursing, caregivers, quality of life, mental health, complex chronic disease

## Abstract

Background: informal caregivers have a high risk of suffering from diseases derived from the chronic stress to which they are subjected for their dedication to the care of their relatives. Such stress has a direct influence on the person cared for, mainly affecting the quality of their care. Therefore, the aim of the present study is to assess the association of caregiving on physical and mental perceived health in family caregivers of dependent adults with complex chronic diseases. Methods: a prospective longitudinal cohort study, with a follow-up period of 36 months (HUELLA cohort). The exposed cohort will be formed by family caregivers of dependent patients with complex chronic pathologies. The unexposed cohort will be taken from the general population adjusted for age, sex and health. Outcome variables will include attendance to health services, consumption of psychoactive drugs, dedication to care (only in exposed cohort), concession of the Act on Promotion of Personal Autonomy and Care for dependent persons (exposed only), perceived physical and mental health, depression level, burden level and new diagnosis of chronic pathology of the caregiver during the study. Results: the expected results will be applicable and will incorporate improvements to the usual health system clinical practice, providing feedback to professionals dedicated to the provision, planning and design of services to family caregivers, as well as to groups and organizations of caregivers. Conclusions: investments in preventing low-quality informal care are key, mainly through early identification and interventions to support caregivers who suffer from stress, anxiety or depression.

## 1. Introduction

Informal care refers to unpaid care provided by family members, friends and charities to individuals in need of help with everyday tasks [1,2].

Caregivers use their own health reserve to care for their relatives, which becomes a risk factor that can interfere with their physical and mental health, as well as their quality of life, mainly due to the overload of care and the deterioration of their social support network [3]. It is common to find problems such as depression, anxiety, social isolation and worse perceived mental health [4,5]. In addition, women with a low education level and who live with the person have a greater risk of overload, depression and social isolation [6].

In Spain, the analysis of the population-based National Health Survey showed a clear disadvantage in the mental health of female carers, with 1.3 times more probability of depression and less perceived social support, although there were no differences in the development of healthy lifestyles [7]. These results correspond to the conceptual framework that has usually been used for analysis of the impact of family care, such as coping with stress of Lazarus and Folkman [8] and the physiological impact of prolonged stress [9].

In terms of physical health, several studies have reported an increased risk of mortality associated with family care, as detected in the classic Schulz study [10], or the incidence of cardiovascular diseases, although it has been evaluated only in certain types of caregivers and seems to be associated with a history of worse perceived health [11,12].

There is an overabundance of cross-sectional and longitudinal studies on the impact of family care on the health of caregivers worldwide [13,14]. In Spain, there have been also cross-sectional [7,15,16,17] and longitudinal studies that have tried to evaluate the association between family care and the impact on health [18,19] but there is no cohort of caregivers that has been followed for more than 12 months and with follow-up periodicities at reasonable intervals.

Therefore, we intend to follow up a sample of caregivers with sufficient elements of methodological robustness in order to shed clarity about the uncertainty that still persists about the ‘mark’ (‘huella’ in Spanish) left by informal health care on those who carry out this social function that sustains an important pillar of the well-being of our country. The aim of the study is to know the effect of care on the physical and mental perceived health of family caregivers of dependent adults and with complex chronic diseases (HUELLA cohort).

## 2. Materials and Methods

### 2.1. Aim

The aim of this study is to know the effect of care on the physical and mental perceived health of family caregivers of dependent adults and with complex chronic diseases (HUELLA cohort).

Specific aims:

Primary

1. To analyze the association between exposure to family care and perceived physical health.

2. To analyze the association between exposure to family care and perceived mental health.

3. To analyze the association between exposure to family care and the level of depression.

Secondary

1. To analyze the association between exposure to family care and the use of health services (primary care and specialized care).

2. To identify characteristics of caregivers and the provision of care associated with the improvement or worsening of perceived health (physical and mental).

### 2.2. Design

Prospective longitudinal cohort study. We have chosen this type of study to respond to our aim, because there is no cohort study of caregivers followed-up for more than 12 months and with follow-up periodicities at reasonable intervals. By measuring outcomes such as their perceived mental and physical health over a period of time and comparing these results with those obtained in the control group, it will allow us to explore the impact of caregiving in those variables. Our reporting adheres to STROBE (The Strengthening the Reporting of Observational studies in Epidemiology) guidelines.

### 2.3. Methodology

#### 2.3.1. Sample/Participants

Our research group has carried out three cross-sectional studies with different aspects of mental health and quality of life of caregivers of dependent patients with complex chronic diseases, with a total sample of 1027 caregivers. The exposed cohort of our study will be formed by family caregivers of dependent patients and/or with complex chronic diseases, treated in the health districts of Malaga and “Costa del Sol”, both at primary and hospital levels, recruited from three previous studies: EMPADEC [20], ECCUPENIC [21], and TALISMAN. The unexposed cohort will be taken from the general population adjusted by age, sex and health matching those belonging to the exposed cohort. Characteristics of these studies were:

1. ECCUPENIC [20]: Case-control study nested in a cohort formed of patients with heart failure admitted to the hospital due to exacerbation, with an identified primary family caregiver, which evaluated the relationship between acute service attendance and Health-Related Quality of Life (HRQOL) in patients and caregivers.

2. EMPADEC [21]: Descriptive longitudinal study to follow up a cohort of family caregivers which analyzed their mental health, quality of life and use of health care services.

3. TALISMAN: Analytical cross-sectional study that included family caregivers of patients whose level of dependency was total, severe or moderate. The aim was to describe the time dedicated to unpaid caregiving work, the eligibility of the caregiver role and the simultaneous dedication to paid work, and also to analyze the relationship between family care time, the presence of paid work and the physical and emotional health of family caregivers.

#### 2.3.2. Inclusion/Exclusion Criteria

Exposed cohort

Family caregivers, with an age over 18 years old, who were selected in their respective studies of origin and who culminated them. We will exclude those subjects from the origin samples who present incomplete data or who do not give their consent to participate in this study. If they have died, they will be excluded if there are no follow-up data available (in some of the three studies, there were longitudinal follow-ups) or in case the last follow-up has been made far from the date of death (more than 12 months). Caregivers will also be excluded if, at the time of their inclusion in their original study, they had already presented some of the following comorbidities: schizophrenia, bipolar disorder, posttraumatic stress disorder, eating disorders, addictive and substance use disorders; oncological disease with palliative treatment; heart failure (class IV according to the New York Heart Association (NYHA)); respiratory disease (chronic obstructive pulmonary disease (COPD) at severe stage, pulmonary fibrosis); neurological diseases (demyelinating, motor neuron, dementia, Parkinson’s in advanced stages, neurodevelopmental disorders); cirrhosis; morbid obesity; functional disability that prevents them to do the activities of daily life correctly: Barthel < 60.

Unexposed cohort

The unexposed cohort will include people belonging to the same Primary Health Centers of the exposed cohort by means of random stratified sampling by center, sex and age group, with corresponding matching with a reference group of the exposed cohort. It will include subjects who have never been a family caregiver of a person with medium or high level of dependence or multimorbidity, or of children with disabilities or some chronic process or degenerative disease, or of people with psychiatric incapacitating morbidity, and who give their consent to participate in the study during the corresponding follow-up periods. The following will be excluded:

Subjects with a cognitive impairment or intellectual disability that prevents them from completing the questionnaires and data collection instruments, or those that, for linguistic reasons, do not have sufficient fluency for this purpose; subjects who became family caregivers during the study; subjects who have a professional health or social care activity (medical doctors, nurses, laboratory technicians, auxiliary nursing technicians, social workers, professional caregivers, occupational therapists); subjects who, at the time of selection, present some of the comorbidities described in the exclusion criteria section of the exposed cohort.

#### 2.3.3. Sample Size

For the sample size calculation, three large studies that have longitudinally evaluated the association of caring on perceived health and the level of depression in family caregivers have been chosen as a reference, in order to establish a calculation framework as close as possible. In this sense, to detect a minimum relative risk of 1.21 in the perception of physical health and of 1.46 in the level of depression, [13], with an alpha of 0.05 and a statistical power of 80%, we will require 913 subjects per group (total 1826) in the first case, and 348 per group (total 696) in the second.

On the other hand, to detect a minimum relative risk of 1.16 in the perception of physical health and of 1.60 in the level of depression [17], with the same confidence and power levels, we will require 792 subjects per group (total 1584) in the first case and 78 per group (total 156) in the second.

Taking into account the differences in the mental and physical health components of the Short Form-12 Health Survey (SF-12) between caregivers and non-caregivers, in order to detect a difference of 2.1 points in the physical health component with an alpha level of 0.05 and a statistical power of 80%, 471 subjects per group (total 942) will be needed. In addition, for a difference of 1.17 points in the mental health component [3], 689 subjects per group are necessary (1378 in total). Therefore, with 1826 subjects, both sample needs would be covered.

Finally, as we have baseline data on the perceived health of the exposed cohort, an estimate has been made with respect to the reference data in the Spanish population of the SF-12 and its components [22], in the group of women aged from 55 to 64 (which covers the mean age of the exposed sample cohort). Thus, to detect a difference of 5.7 points in the mental health component, with the same confidence and power parameters, 156 subjects would be needed; in the case of the physical health component, for a difference of 2.9 points, 476 subjects would be needed.

Therefore, in view of the three estimates, with a cohort of 871 unexposed subjects, the sample needs would be met (the cohort of subjects exposed has 1027 people), with an overestimation of 10% to cover possible losses (there would be almost 42 subjects of difference with the first estimated sample scenario, which does not use the same measurement instruments).

### 2.4. Follow-up Period

Follow-ups will be carried out every 6 months throughout the three years of the study. All the data will be entered in a database in a segregated form so that only the Unique Health History Number of Andalusia (NUHSA, as the Spanish acronym) will be included as the only identifying data.

### 2.5. Instruments and Variables

Prior to the beginning of the study, we proceeded to perform an evaluation of the variables present in the databases of each study of origin to match them and obtain a final corpus of common variables in the three studies. Those variables that did not have a homogeneous classification system in the three studies were also recoded with a common format:

- Sociodemographic variables: Age of caregiver; age of the person cared for; sex of the caregiver; sex of the person cared for; occupation and level of education of the caregiver; marital status and dependency level of the person cared for, measured with the Barthel Dependency Scale (only in the exposed cohort).
- Attendance to health services variables: hospital admissions during follow-up; emergency visits during the period; visits to the primary care physician; visits to the family nurse and visits to hospital specialists. This will be consulted in their medical record, using DIRAYA, the system used by the Andalusian Health Service to support electronic medical records.
- Consumption of psychoactive drugs: This will be consulted in their medical record, using DIRAYA.
- Dedication to care (only in the exposed cohort): Daily dedication to care (hours) and duration of care (months).
- Concession of the Act on Promotion of Personal Autonomy and Care for dependent persons (exposed only): This was part of a law (included in the Spanish Official State Bulletin, Law 39/2006 of December 14 of Promotion of Personal Autonomy and Attention to People in Dependency Situation), that granted social and healthcare assistance to both the caregiver and the cared person, and also an economic benefit to the caregiver.
- Caregiver outcomes: Perceived physical and mental health, measured with the SF-12 Questionnaire [23,24]; depression level, measured with the Patient Health Questionaire-9 (PHQ9) [25]; burden level, measured with the Caregiver Effort Index (CEI) [26,27].
- New diagnosis of chronic pathology of the caregiver during the study: This will be consulted in their medical record, using the Andalusian Health Service health history (DIRAYA).

#### 2.5.1. Data Collection

The recruitment of the samples will be done by telephone, as well as the data collection by accessing to subjects’ Andalusian Health Service health history. All the questionnaire data will be also collected by telephone.

#### 2.5.2. Data Analysis

The analyses will be carried out blindly by an evaluator outside the research team.

Descriptive and exploratory analysis:

Descriptive statistics of the variables will be carried out by obtaining measures of central tendency and dispersion or percentages, according to the nature of them. Normality of the distribution of all variables will be assessed by a Kolmogorov–Smirnov test, histograms, asymmetry and kurtosis.

Bivariate analysis:

Contrast tests will be made by Chi-square test and Mantel–Haenszel statistics, with Fisher’s exact correction if necessary, in qualitative variables. In all the parameters, accuracy will be estimated by calculating 95% confidence intervals. For continuous variables, bivariate analysis will be performed by a Student’s t test for independent samples if they present a normal distribution. In the case of a distribution different from normal, nonparametric tests (Mann–Whitney U and Wilcoxon test) will be used. Group analyses will also be carried out by means of ANOVA (after checking homogeneity of variances with the Levene test) and post-hoc analysis by Bonferroni’s test or robust tests (Welch and Hosmer–Lemeshow) in case of non-homoscedasticity. If the normality of the distributions prevents these calculations, the Kruskal–Wallis test will be performed. The sample will be stratified according to the differential values of the main variables of Mental Health, Overload, Quality of Life and Utilization of Services, as well as sociodemographic determinants (age, sex, level of studies, etc.) in order to identify possible baseline differences and explore possible confusion effects or modification of effect, and take them into account in the subsequent calculations through corresponding adjustments. Kaplan–Meyer analyses will be performed with a log-rank test to estimate differences in outcomes, presence of overload (according to validated cut-off points in the Spanish population), presence of depression (according to validated cut-off points) and physical and mental health perceived to be below the cutoff point of the Spanish population. For the analysis of the intrasubject changes in scores of PHQ9, SF12 and CEI, general linear models will be made for repeated measures with a Bonferroni penalty.

Multivariate analysis:

The hypothesis contrast will be carried out by estimating the hazard ratio (HR) of SF-12 and PHQ9 through a Cox regression model of proportional hazards, taking the time until the event as the interval from introduction into the cohort to the emergence of the outcome, according to the cutoff points validated in the population. Raw and adjusted HRs will be calculated by introducing individual variables to the model that modify the result by 10% or more and eliminating those that do not show significant statistical association. Generalized linear models will be also made for the analysis of quantitative differences in the values of the mental and physical health component of the SF-12, as well as the PHQ9.

### 2.6. Ethical Considerations

The study is authorized by a Research Ethics Committee. The standards of good clinical practice and the ethical principles established for human research in the Declaration of Helsinki and its subsequent revisions will be maintained at all times.

## 3. Discussion

Informal care is often considered a cost-effective way to prevent institutionalization and allow users to remain in their homes [28,29]. However, informal care is not free for people or for the state. The needs of caregivers and the impact of providing informal care on key life outcomes, such as employment, health and well-being, are increasingly recognized in academic literature and national policies throughout Europe [5]. The European Pillar of Social Rights makes explicit the commitment to the people who provide care, including their rights to flexible work and access to care services. In this sense, in the context of cost containment versus the growing need for care, the provision of family care remains key [13], being considered crucial to avoid unnecessary and costly hospitalization or institutionalization. All this implies that investments in preventing low-quality informal care are key, mainly through early identification and interventions to support caregivers, and could prevent them suffering from stress, anxiety or depression [3,6,30].

Our research group has carried out three previous studies in the past ten years involving caregivers, where we studied the effect of caring for their beloved ones on their physical and mental health and quality of life, whose results showed, among others, the relationship between the use of hospital services and a worse perceived quality of life of both patients and their family caregivers [31]. Nonetheless, none of those studies had sufficient elements of methodological robustness due to the control groups as well as sample sizes. The expected results of this research will be applicable and will incorporate improvements in the usual clinical practice of the health system, since it will serve to provide feedback to professionals who provide care for people with a complex chronic disease in the social and health sectors, and will help to redirect the social-health care that informal caregivers receive in our National Health System.

### Limitations

First of all, in any cohort study, the possibility of sample losses during follow-ups should not be ignored. The sample has been overestimated by 10% and, in addition, in the exposed cohort, there is a sufficient sample volume that would compensate for losses of up to 30%. In addition, a telephone contact system will be established, with frequent recall calls and with the collaboration of nurse case managers from the primary health centers that would act as mediators in any cases of difficult follow-up. The method of collecting data through telephone contact implies a loss of contact with the subject compared to a personal interview. This could result in an increase in subjects losses during the study, as well as difficulty in understanding the questionnaires used.

## 4. Conclusions

This study lays the foundations of a cohort of informal caregivers nationwide that will be key to understanding their quality of life and the relationship it has with the act of caring.

The expected results are applicable and will incorporate improvements in the usual clinical practice of the Health System since they will serve to provide feedback to the professionals dedicated to the provision, planning and design of services for family caregivers, as well as to groups and organizations of caregivers and mutual aid groups. This information can be used to offer feedback to professionals who provide care for caregivers that care for people with complex chronic diseases. The knowledge and transfer of this information will help the reorientation of the social-health care received by informal caregivers in our National Health System and may be an additional contribution to the assessment criteria of family support in dependency situations.

## References

[1] Costa-Font J., Karlsson M., Øien H. (2016). Careful in the Crisis? Determiinants of Older People’s Informal Care Receipt in Crisis-Struck European, Countries. Health Econ..

[2] Genet N., Boerma W.G.W., Kringos D.S., Bouman A., Francke A.L., Fagerström C., Melchiorre M.G., Greco C., Devillé W.L.J.M. (2011). Home care in Europe: A systematic literature review. BMC Health Serv. Res..

[3] Roth D.L., Perkins M., Wadley V.G., Temple E.M., Haley W.E. (2009). Family caregiving and emotional strain: Associations with quality of life in a large national sample of middle-aged and older adults. Qual. Life Res..

[4] Del Río-Lozano M., García-Calvente M.D.M., Marcos-Marcos J., Entrena-Durán F., Maroto-Navarro G. (2013). Gender Identity in Informal Care. Qual. Health Res..

[5] Verbakel E., Tamlagsrønning S., Winstone L., Fjær E.L., Eikemo T.A. (2017). Informal care in Europe: Findings from the European Social Survey (2014) special module on the social determinants of health. Eur. J. Public Health.

[6] Adelman R.D., Tmanova L.L., Delgado D., Dion S., Lachs M.S. (2014). Caregiver burden: A clinical review. JAMA.

[7] Paz L.G.-D., Real J., Borrás-Santos A., Martínez-Sánchez J.M., Rodrigo-Baños V., Navarro-Rubio M.D., Paz J.R.L.G.-D. (2016). Associations between informal care, disease, and risk factors: A Spanish country-wide population-based study. J. Public Health Policy.

[8] Lazarus R.S., Folkman S. (1984). Stress, Appraisal, and Coping.

[9] Hawken T., Turner-Cobb J., Barnett J. (2018). Coping and adjustment in caregivers: A systematic review. Health Psychol. Open.

[10] Schulz R., Beach S.R. (1999). Caregiving as a Risk Factor for Mortality. JAMA.

[11] Buyck J.-F., Ankri J., Dugravot A., Bonnaud S., Nabi H., Kivimäki M., Singh-Manoux A. (2013). Informal Caregiving and the Risk for Coronary Heart Disease: The Whitehall II Study. J. Gerontol. Ser. A Biomed. Sci. Med. Sci..

[12] Ji J., Zöller B., Sundquist K., Sundquist J. (2012). Increased Risks of Coronary Heart Disease and Stroke Among Spousal Caregivers of Cancer Patients. Circulation.

[13] Hiel L., Beenackers M., Renders C.M., Robroek S.J., Burdorf A., Croezen S. (2015). Providing personal informal care to older European adults: Should we care about the caregivers’ health?. Prev. Med..

[14] Vlachantoni A., Evandrou M., Falkingham J., Robards J. (2013). Informal care, health and mortality. Maturitas.

[15] Abajo M., Rodríguez-Sanz M., Malmusi D., Salvador M., Borrell C. (2016). Gender and socio-economic inequalities in health and living conditions among co-resident informal caregivers: A nationwide survey in Spain. J. Adv. Nurs..

[16] Del-Pino-Casado R., Palomino-Moral P.A., Frías-Osuna A. (2015). The Association of Satisfaction and Perceived Burden with Anxiety and Depression in Primary Caregivers of Dependent Elderly Relatives. Res. Nurs. Health.

[17] Salvador-Piedrafita M., Malmusi D., Borrell C. (2017). Time trends in health inequalities due to care in the context of the Spanish Dependency Law. Gac. Sanit..

[18] Conde-Sala J.L., Turrò-Garriga O., Calvó-Perxas L., Vilalta-Franch J., López-Pousa S., Garre-Olmo J. (2014). Three-Year Trajectories of Caregiver Burden in Alzheimer’s Disease. J. Alzheimer Dis..

[19] Romero-Moreno R., Márquez-González M., Mausbach B.T., Losada A. (2012). Variables modulating depression in dementia caregivers: A longitudinal study. Int. Psychogeriatr..

[20] Cuevas-Fernández-Gallego M., Morales-Asencio J.M., Martín-Santos F.J., Arándiga R.C., Contreras-Fernández E., Sicilia J.P.B., Navarro-Moya F.J., Abajo I.L., Celdrán-Mañas M., Bonill-De-Las-Nieves C. (2012). Effect of the act on promotion of personal autonomy and care for dependent persons on their family caregivers. BMC Health Serv. Res..

[21] Timonet-Andreu E.M., Morales-Asencio J.M., Canca-Sánchez J.C., Sanchez J.S., Rico R.M., Rivas-Ruiz F. (2015). Effects and consequences of caring for persons with heart failure: (ECCUPENIC study) a nested case-control study. J. Adv. Nurs..

[22] Schmidt S., Vilagut G., Garin O., Cunillera O., Tresserras R., Brugulat P., Mompart A., Medina A., Ferrer M., Alonso J. (2012). Normas de referencia para el Cuestionario de Salud SF-12 versión 2 basadas en población general de Cataluña. Med. Clín..

[23] Monteagudo P.O., Hernando A.L., Palomar R.J.A. (2011). Population based norms of the Spanish version of the SF-12V2 for Murcia (Spain). Gac. Sanit..

[24] Orive M., Aguirre U., García-Gutiérrez S., Hayas C.L., Bilbao A., Gonzalez N., Zabala J., Navarro G., Quintana J.M. (2015). Changes in health-related quality of life and activities of daily living after hip fracture because of a fall in elderly patients: A prospective cohort study. Int. J. Clin. Pract..

[25] Pinto-Meza A., Serrano-Blanco A., Peñarrubia-María M., Blanco E., Haro J.M. (2005). Assessing depression in primary care with the PHQ-9: Can it be carried out over the telephone?. J. Gen. Intern. Med..

[26] Hoffmann T., Worrall L., Eames S., Ryan A. (2010). Measuring Outcomes in People Who Have Had a Stroke and Their Carers: Can the Telephone Be Used?. Top. Stroke Rehabil..

[27] Nejad Z.K., Aghdam A.M., Hasankhani H., Sanaat Z. (2016). The Effects of a Patient-Caregiver Education and Follow-Up Program on the Breast Cancer Caregiver Strain Index. Iran. Red Crescent Med. J..

[28] Dicker D., Nguyen G., Abate D., Abate K.H., Abay S.M., Abbafati C., Abbasi N., Abbastabar H., Abd-Allah F., Abdela J. (2018). Global, regional, and national age-sex-specific mortality and life expectancy, 1950–2017: A systematic analysis for the Global Burden of Disease Study 2017. Lancet.

[29] Vetrano D.L., Palmer K., Marengoni A., Marzetti E., Lattanzio F., Roller-Wirnsberger R., Samaniego L.L., Rodríguez-Mañas L., Bernabei R., Onder G. (2018). Frailty and Multimorbidity: A Systematic Review and Meta-analysis. J. Gerontol. Ser. A Biomed. Sci. Med. Sci..

[30] Roth D.L., Fredman L., Haley W.E. (2015). Informal Caregiving and Its Impact on Health: A Reappraisal from Population-Based Studies. Gerontologist.

[31] Timonet-Andreu E.M., Morales-Asencio J.M., Gutierrez P.A., Alvarez C.C., Rn G.L., Banderas A.M., López-Leiva I., Canca-Sanchez J.C. (2020). Health-Related Quality of Life and Use of Hospital Services by Patients with Heart Failure and Their Family Caregivers: A Multicenter Case-Control Study. J. Nurs. Sch..

